# Associations between smoking and blood-group, and the risk of dyslipidaemia amongst French women

**DOI:** 10.1038/s41598-021-94239-9

**Published:** 2021-07-21

**Authors:** C. J. MacDonald, A. L. Madika, G. Severi, A. Fournier, M. C. Boutron-Ruault

**Affiliations:** 1 INSERM (Institut National de La Santé Et de La Recherche Médicale) U1018, Center for Research in Epidemiology and Population Health (CESP), Institut Gustave Roussy, 114, rue Edouard Vaillant, 94805 Villejuif cedex, France; 2 Université Paris-Saclay, Université Paris-Sud, Villejuif, France; 3 Université de Lille, CHU Lille, EA 2694 - Santé Publique : Épidémiologie et Qualité des Soins, 59000 Lille, France; 4 Department of Statistics, Computer Science and Applications ”G. Parenti”, University of Florence, Florence, Italy

**Keywords:** Epidemiology, Cardiovascular biology

## Abstract

Dyslipidaemia is a major risk factor for cardio-vascular disease, as it promotes atherosclerosis. While cross-sectional studies have identified higher serum cholesterol amongst individuals with the A blood group, there is less evidence from prospective studies whether this translates into a higher risk of dyslipidaemia that requires treatment, nor if this genetic factor interacts with smoking status. This study aimed to prospectively determine potential associations between smoking, ABO blood groups, and risk of incident dyslipidaemia requiring treatment, and to assess associations over strata of blood ABO group. We assessed associations between blood ABO group, smoking and dyslipidaemia in 74,206 women participating in the E3N cohort. We included women who did not have cardiovascular disease at baseline. Logistic regression was used to determine associations between ABO group, smoking and prevalent dyslipidaemia at baseline. Cox proportional hazard models were then used to determine if blood ABO group and smoking were associated with the risk of incident dyslipidaemia, amongst women free of dyslipidaemia at baseline. At baseline 28,281 women with prevalent dyslipidaemia were identified. Compared to the O-blood group, the non-O blood group was associated higher odds of with prevalent dyslipidaemia (OR_non-O_ = 1.09 [1.06: 1.13]). Amongst the women free of dyslipidaemia at baseline, 6041 incident cases of treated dyslipidaemia were identified during 454,951 person-years of follow-up. The non-O blood groups were associated with an increased risk of dyslipidaemia when compared to the O-group (HR_non-O_ = 1.16 [1.11: 1.22]), specifically the A blood-group (HR_A_ = 1.18 [1.12: 1.25]). Current smokers were associated with an increased risk of incident dyslipidaemia (HR _smokers_ = 1.27 [1.16: 1.37]), compared to never-smokers. No evidence for effect modification between smoking and ABO blood group was observed (p-effect modification = 0.45), although the highest risk was observed among AB blood group women who smoked (HR = 1.76 [1.22: 2.55]). In conclusion, the non-O blood groups, specifically the A group were associated with an increased risk of dyslipidaemia. Current smokers were associated with a 30% increased risk of dyslipidaemia. These results could aid in personalised approaches to the prevention of cardiovascular risk-factors.

## Introduction

Dyslipidaemia is the main risk-factor for heart disease^1^, and can lead to the formation of atherosclerotic plaques^2^ which can narrow the coronary arteries, causing life-threatening blockages. High low-density lipoprotein (LDL) cholesterol levels can also lead to endothelial dysfunction and oxidative stress^3^, both additional risk-factors for heart disease^4^.

The A blood group has been found to be associated with higher serum cholesterol levels^5^ since the observation of higher cholesterol among non-O blood group males in the prospective Framingham Heart study of 5209 adults, and amongst 10,000 Israeli government employees in the 1970s^6, 7^, and it has been suggested that associations between non-O groups and cardiovascular disease are mediated by serum cholesterol levels^8^. The non-O blood groups, most commonly the A-group, have been identified as a genetic risk factor for cardiovascular disease in meta-analyses of prospective and retrospective studies^9–11^ and have long been associated with higher rates of ischemic heart disease. Similarly, smoking is a major risk-factor for dyslipidaemia^12, 13^ and further cardiovascular disease^14^, Current smokers have been shown to have higher serum cholesterol levels than non-smokers, including higher low-density lipoprotein (LDL) levels, but also low high-density lipoprotein (HDL) concentrations^15, 16^. Smoking is also associated with increased oxidation of LDL cholesterol, which may explain associations with atherosclerosis^7^, and it has been shown to interact with ABO blood group in other vascular diseases including thromboembolism^17^. Studies have not assessed if interactions with blood group are present when considering dyslipidaemia, despite both factors leading to abnormal lipid profiles. If current smokers with non-O blood group were found to be at an even higher risk of dyslipidaemia, this could aid in the search for personalised preventative approaches for cardiovascular risk factors.

We aimed to investigate if non-O blood group and smoking were associated with an increased risk of dyslipidaemia in a large prospective cohort of French women, and to investigate if there was evidence for effect modification between the two risk-factors, hypothesising that stronger associations may be observed among non-O group women who smoked, due to higher lipid levels.

## Methods

All methods were carried out in accordance with the relevant guidelines and regulations.

### The E3N cohort

The Etude Épidémiologiquede femmes de la Mutuelle Générale de l´Education (E3N) is a French prospective cohort started in 1990 comprising 98,995 women aged 40–65 years at baseline and insured by the Mutuelle Générale de l´Education Nationale (MGEN), a health insurance plan for employees of the French education system and their families^18^. The cohort received ethical approval from the French National Commission for Computerized Data and Individual Freedom (Commission Nationale Informatique et Libertés), and all participants in the study signed an informed consent form. Participants returned mailed questionnaires on lifestyle information and disease occurrence every 2 to 3 years (1990, 1992, 1993, 1995, 1997, 2000, 2002, 2005, 2008, 2011, and 2014), resulting in 11 questionnaires (Q1-11). The average response rate at each questionnaire cycle was 83%, and the total loss to follow-up was 3%. Furthermore, for each participant, the MGEN health insurance plan provided data that included all drug reimbursements since January 1, 2004.

### Assessment of dyslipidaemia

In the first two questionnaires sent in 1990 and 1992 (Q1 & Q2), participants were asked if they were undergoing treatment for raised blood cholesterol, and to provide serum cholesterol values if available in mmol/L. In Q6, Q7 and Q8 participants were asked if their previous cholesterol measurement was abnormal, and to provide serum cholesterol values. In Q9, Q10 and Q11, participants were asked to report if they were undergoing treatment for high cholesterol.

The drug reimbursement database allowed us to identify reimbursement for lipid lowering medications in 2004 (identified using Anatomical Therapeutic Chemical Classification System C10A and C10B). We considered incident cases of dyslipidaemia as those reporting high or treated blood cholesterol, confirmed by a reimbursement for lipid lowering medications at the same time as the questionnaire (+−1 year, except for Q6 which considered reimbursements at 2004).

Cases in this study were defined as either: self-reported ‘abnormal or high cholesterol requiring treatment’, or self-reported serum cholesterol >  = 6.6 mmol / L (‘self-reported dyslipidaemia’). We considered an additional criteria, requiring identification of a lipid-lowering medication during the period corresponding to the questionnaire that dyslipidaemia was reported (‘treated dyslipidaemia’). Due to the significant number of women not reporting cholesterol values, we did not consider cholesterol in mmol / L as the outcome.

### Assessment of blood ABO group

Participants were asked to report their blood ABO group in the first questionnaire as *O*, *A*, *B*, or *AB*, as well as their Rhesus group as positive or negative.

### Assessment of smoking status

Smoking status was considered at every questionnaire, and was based on self-reports. Subjects were classified as never, former or current smokers. Past or current smokers of less than one cigarette per day (occasional smokers) were considered non-smokers.

### Assessment of covariates

Height and weight were self-reported at each questionnaire and used to calculate body mass index (BMI (kg/m^2^)). Family history of cardiovascular disease (stroke or myocardial infarction) was based on self-reports. Education level was self-reported and used as a proxy for social class. Total physical activity was self-reported in Q5, and detailed time spent undergoing various activities (such as walking, housework, sports). Total Metabolic equivalents (MET-hours) were estimated for each individual using the compendium of physical activity. Hypertension was self-reported and verified by cross-correlation with the drug reimbursement database, to confirm that the participants were undergoing treatment. Use of menopausal hormone therapy (MHT) was self-reported during follow-up. The complete history of HT use was established using data from all the questionnaires^19^. Among postmenopausal women, age at menopause was defined as either (in decreasing order of priority) age at last menstrual period, age at bilateral oophorectomy, self-reported age at menopause, age at start of MHT, or the age at the start of menopausal symptoms. If unavailable, the median age at menopause for the cohort (51 years for natural menopause, 47 years for artificial menopause) was imputed.

### Study population

From the 98,995 women of the E3N study, we excluded women for whom there was no information on potential dyslipidaemia (n = 14,652), blood group (n = 7189), at or prior to Q6, women who died, or did not provide data on covariates prior to Q7 (N = 2948), resulting in 74,206 women being included.

### Statistical analysis

Two main analyses were performed. We first considered associations between prevalent self-reported dyslipidaemia at baseline (Q7) and the characteristics of the cohort, including blood ABO group. Secondly, we considered only incident cases after Q7 in a time-to-event model, and excluded all prevalent cases considered in the first analysis.

#### Baseline associations

We assessed associations between baseline variables at Q7 and self-reported prevalent dyslipidaemia (yes/no) amongst women alive at Q7 using a logistic regression model, with prevalent dyslipidaemia as a binary variable. Results were interpreted as odd ratio for prevalent dyslipidaemia, with 95% confidence interval.

#### Incident dyslipidaemia cases

For the analysis of incident dyslipidaemia cases, we considered a time-to-event model, and excluded all prevalent cases from this analysis, resulting in a sample of 45,925. Cases were initially considered as all incident self-reports of abnormal blood cholesterol, and then only self-report verified reimbursement for lipid-lowering medications. Participants contributed person years to the analysis from inclusion until the participant reported dyslipidaemia, the participant’s death, loss to follow up, or the administrative end of the study period (June 2014).

ABO group was considered an exposure in Cox proportional hazard models, with the O-group as reference. ABO group was considered as *O*/*non-O*, then as *O*, *A*, *B*, and *AB,* and also Rhesus + or −. Smoking status at baseline was then considered as the exposure, grouped as current smoker, ex-smoker, and never smokers, with never smokers as the reference group.

Models were initially adjusted with age as the timeline, followed by potential confounders BMI (continuous), physical activity (continuous), education (university degree or not), treated hypertension (yes/no), treated diabetes (yes/no), family history of CVD (yes/no), ever use of menopausal hormone therapy (yes/no), age at menopause. We assessed a-priori effect-modification between ABO group and smoking status by including the joint term in the main model. The p-value for this term was used to determine if effect-modification was likely.

Data is presented as mean (standard deviation) for continuous variables, median (inter-quartile range) for non-normal data and % for categorical variables. All analysis was conducted using the statistical software R, version 3.5, with the survival package.

### Ethical approval and consent to participate

The cohort received ethical approval from the French National Commission for Computerized Data and Individual Freedom (Commission Nationale Informatique et Libertés), and all participants in the study signed an informed consent.

## Results

At inclusion, there were 70,177 women included in the study. *Non-O* blood group was reported in 56.7% of women. The mean age was 59.2 years old, BMI was 23.8 kg/m^2^, and 90.5% of women were menopausal. Full cohort characteristics are presented in Table 1.

**Table 1:** Associations between baseline characteristics and prevalent dyslipidaemia, odds ratio calculated using a mutually adjusted logistic regression model.

	Whole population N = 74,206	No dyslipidaemia N = 45,925	Prevalent dyslipidaemia N = 28,281	Odds ratio between no dyslipidaemia and prevalent dyslipidaemia*
Cholesterol (mmol/L)	5.8 (1.1)	5.4 (0.8)	6.2 (1.3)	–
Missing data on serum-cholesterol (%)	42.9	48.4	34.1	–
Reimbursed for lipid-lowering medication (%)	26.0	14.8	44.1	–
Cohort characteristics				
Never smoker (%)	53.6	52.6	55.4	Ref
Ex-smoker (%)	36.9	37.1	36.5	1.00 [0.97: 1.03]
Current smoker (%)	9.5	10.3	8.1	0.87 [0.82: 0.92]
Non-O blood group (%)	56.8	55.9	58.3	1.09 [1.06: 1.13]
Rhesus group + (%)	82.9	83.0	82.6	0.98 [0.94: 1.03]
Age at baseline (years)	61.5 (6.5)	60.7 (6.3)	62.7 (6.6)	1.05 [1.04: 1.05]
BMI (kg/m^2^)	23.8 (3.7)	23.7 (3.7)	24.0 (3.7)	1.01 [1.00: 1.02]
Total physical activity (Mets-h/week)	65.6 (37.8)	61.7 (37.6)	63.1 (38.0)	1.00 [1.00: 1.00]
Family history of cardiovascular disease (%)	34.7	32.6	38.2	1.30 [1.25: 1.34]
Prevalent hypertension (%)	43.4	41.1	47.1	1.11 [1.07: 1.14]
Prevalent diabetes (%)	3.4	2.9	4.1	1.27 [1.16: 1.38]
Education (high-school or higher)	87.6	88.1	86.7	0.98 [0.93: 1.02]
Menopausal at baseline	89.6	88.0	92.2	1.07 [1.00: 1.15]
Ever use of MHT **(yes or no)	60.0	60.0	60.1	0.99 [0.96: 1.03]
Age at menopause **(years)	50.3	50.6	50.4	0.99 [0.98: 0.99]

*Adjusted simultaneously for all variables.

**Amongst menopausal women.

*BMI* body mass index, *MHT* menopausal hormone therapy.

### Associations between prevalent dyslipidaemia and baseline characteristics

At baseline 17,223 women reported prevalent dyslipidaemia. Cholesterol values were provided by 42,358 (57.1%) participants, and values were higher among women reporting dyslipidaemia, compared to those who did not (6.2 vs 5.4 mmol/L). Among women reporting dyslipidaemia, cholesterol values were on average lower if they were reimbursed for lipid–lowering drugs (6.4 vs 5.9 mmol/L). Cholesterol values were similar among women not reporting dyslipidaemia, regardless of statin reimbursement (data not shown).

Using a multivariate logistic regression model, it was observed that those with prevalent dyslipidaemia were more likely to be of *non-O* blood group (OR = 1.17 [1.13: 1.21], Table 1) compared to those without dyslipidaemia. Women with dyslipidaemia at baseline were also more likely to be older, have a higher BMI, more likely to have familial history of cardiovascular disease, have diabetes or hypertension, and were less likely to be current smokers (Table 1).

### Associations between blood ABO group, and incident dyslipidaemia during follow-up

Amongst the 45,925 women free of dyslipidaemia at baseline, 8370 reported incident high blood cholesterol during 454,951 person years, 6041 (72.2%) of whom were verified by reimbursement for lipid-lowering medications. This corresponded to a rate of 18.4/1,000 PY. No major differences in these women’s characteristics were observed according to blood-group (supplementary Table 1).

Compared to the O group, the non-O groups were associated with an increased risk of incident dyslipidaemia (HR _non-O_ = 1.14 [1.10: 1.19], Table 2). Specifically, the *A* blood group was associated with an increased risk of dyslipidaemia when compared to the O-group (HR_A_ = 1.18 [1.13: 1.23], Table 2), independent of other risk factors. Other blood groups were not associated with the risk of dyslipidaemia (HR_B_ = 1.03 [0.98: 1.09], HR _AB_ = 1.04 [0.97: 1.12], Table 2). Rhesus group was not associated with the risk of dyslipidaemia.

**Table 2:** Risk of incident dyslipidaemia depending on blood ABO group, hazard ratios (HR) estimated from Cox proportional hazards models.

Blood group	Self-reported dyslipidaemia (cases = 8370 )	Self-reported dyslipidaemia + medication reimbursement (cases = 6041)
*O (n* = *20,260)*	Ref	Ref
*Non-O (n* = *25,665)*	1.14 [1.09: 1.19]	1.16 [1.11: 1.22]
*A (n* = *19,899)*	1.15 [0.10: 1.20]	1.18 [1.12: 1.25]
*B (n* = *4020)*	1.06 [0.98: 1.15]	1.08 [0.98: 1.19]
*AB (n* = *1746)*	1.14 [1.02: 1.27]	1.13 [0.98: 1.29]
*Rhesus* + *(n* = *38,118)*	Ref	Ref
*Rhesus – (n* = *7807)*	0.97 [0.92: 1.03]	0.97 [0.91: 1.03]
*Smoking status*		
*Never smoker (n = 24,144)*	Ref	Ref
*Ex-smoker (n = 17,043)*	1.07 [1.02: 1.12]	1.04 [0.99: 1.10]
*Current smoker (n = 4738)*	1.23 [1.14: 1.33]	1.27 [1.16: 1.37]

Adjusted for body mass index, physical activity, education level, diabetes, hypertension, family history of cardiovascular disease, use of menopausal hormone therapy, and age at menopause. Age as the timescale.

Compared to never smokers, women who were current smokers were of an increased risk of incident dyslipidaemia, in both age adjusted, and adjusted models (adjusted HR _smokers_ = 1.33 [1.24: 1.43]; HR _ex-smokers_ = 1.06 [1.01: 1.10], Table 2).

Effect modification was not likely between blood group (O vs non-O) and smoking (p- effect modification = 0.45). Participants with an O blood group who were not smokers reported baseline serum cholesterol values of 5.4 mmol/L at baseline, compared to 5.5 mmol/L among A-group participants who smoked. Associations between current smokers and dyslipidaemia were slightly higher amongst participants with a non-O blood group (HR smokers = 1.31 [1.16: 1.47], Table 3), compared to those with an O blood group (HR smokers = 1.22 [1.06: 1.40], Table 3), although the confidence intervals overlapped. Smokers of the AB-group were at the highest risk of dyslipidaemia (HR smokers = 1.76 [1.22: 2.55]). The adjusted case rate for treated dyslipidaemia amongst AB women who smoked was 23.4 cases/1000 PY, compared to 12.8 cases/1000 PY among O-group women who did not smoke.

**Table 3:** Risk of incident dyslipidaemia depending on smoking status, stratified by blood ABO group, hazard ratios (HR) estimated from Cox proportional hazards models.

Blood group	Adjusted* HR				
	O-Group	Non O-Group (n = 20,260)	A (n = 19,899)	B (n = 4020)	AB (n = 1746)
**Smoking status**					
Never smoker	Ref	Ref	Ref	Ref	Ref
Ex-smoker	0.99 [0.91: 1.08]	1.09 [1.01: 1.17]	1.10 [1.02: 1.17]	1.10 [0.94: 1.28]	1.22 [0.97: 1.54]
Current smoker	1.22 [1.06: 1.40]	1.31 [1.16: 1.47]	1.22 [1.09: 1.36]	1.21 [0.92: 1.58]	1.76 [1.22: 2.55]

*Adjusted for body mass index, physical activity, education level, diabetes, hypertension, family history of cardiovascular disease, use of menopausal hormone therapy, and age at menopause. Age as the timescale. Considering dyslipidaemia validated by medication reimbursements.

## Discussion

In this large prospective study, women of *non-O* blood group, specifically *A*, were at an 18% higher risk of developing dyslipidaemia, regardless of other risk factors including diet, smoking, physical activity, and BMI. At baseline, prevalent dyslipidaemia was also associated with other metabolic conditions, such as hypertension, diabetes, family history of cardiovascular disease, and a higher BMI. Smoking prevalence was lower amongst participants with prevalent dyslipidaemia, suggesting possible attempts to improve lifestyle factors following a diagnosis of high-cholesterol. During follow-up, current smokers were associated with a near 30% increased risk of dyslipidaemia compared to non-smokers. No interaction between blood-group and smoking was observed, although risk was slightly higher amongst smokers with an A blood-group.

Previous large prospective studies conducted since the 1970s^6, 7^, have shown that high concentrations of cholesterol are more common in *non-O* blood group persons and particularly in A-group people. Interestingly, we observed that only the *A*-group was associated with increased rates of dyslipidaemia, and not the *B* or *AB*, although this could be explained by lower sample sizes for these women. Similar results have been observed recently in the large prospective UK biobank study (n = 406 755)^20^, with observations that the *A*-blood group was associated with a 9% higher risk of hyperlipidaemia when compared to the *O*-group, and 8% when compared to the *B*-group. *Non-O* was also a predictor of other diseases such as thrombosis in the UK biobank study^20^, and it was also observed that *non-O* groups were associated reduced longevity, and with lower rates of hypertension. Another recent Italian cross-sectional study of 5063 blood donors found that the *O*-group was more prevalent amongst centenarians, and the authors suggest that this is due to associations with longevity^21^. Another small prospective study identified that *non-O* blood group is associated with clinical and subclinical cardiovascular events^22^. Mechanisms between blood-group and cardiovascular diseases are commonly explained via increases in inflammatory markers^23–25^, von Willebrand factor^26^, and a higher cholesterol burden leading to atherosclerosis, and finally to acute cardiovascular events.

Although the exact mechanism in which the blood group allele causes higher serum cholesterol is unclear, studies have shown that risk alleles in ABO group are associated with higher absorption of cholesterol in the intestines^27^, which may be one mechanism in which serum cholesterol is chronically higher, resulting in a higher lifetime cholesterol burden in non-O group people. The non-O variant is considered independent of other genetic factors which could cause familial hypercholesterolemia^28^, such as mutations in the LDL-receptor gene. The A-blood group has previously been associated with a higher risk of coronary artery disease, likely due to associations with serum cholesterol, and previous work has identified that serum cholesterol is a mediator of this relationship^8^.

We did not observe strong effect modification between smoking and blood-group. Smoking has been identified as a factor which alters the blood lipid profile^7, 15, 16^, and results in increases in LDL-C and decreases in HDL-C. Studies have not assessed differences in cholesterol levels across joint smoking and blood groups, a measurement that could be taken in future studies. We observed no evidence for any statistical effect-modification or interaction, although rates of dyslipidaemia were highest amongst women with an AB blood-group who smoked.

The strengths of this study include its prospective design, large number of participants and cases, control for several potential confounders (although as the exposure of blood group was genetic, and could be considered essentially randomised this changed little). As certain people with high-cholesterol may choose to treat the condition with lifestyle changes before considering drugs, we considered both self-reports and self-reports validated by treatments. Despite lacking laboratory cholesterol measurements in the E3N study, which is a major limitation, we considered self-reported dyslipidaemia requiring treatment validated against the use of lipid-lowering medications from a large drug-reimbursement database. Similarly, certain data such as BMI and smoking were based on self-reported data, but have been shown to be reliable^29^. In this cohort of well educated women, strong agreement has been observed between for example; self-report of hypertension and specific anti-hypertensive medications in other validation studies^30^. Whilst we are confident that the cases considered are true cases of dyslipidaemia, there is likely under reporting in the cohort from undiagnosed cases, which may weaken the observed associations. Other limitations include those common to all observational research; we cannot claim causality, there may be unmeasured confounding, and some participants may have misreported information. For example, unmeasured confounding likely explains that women with dyslipidaemia at baseline were less likely to smoke, and is possibly explained by health seeking behaviour (i.e. quitting smoking) as a result of a diagnosis of dyslipidaemia. Sample sizes in certain subgroups, for example AB women, were small. In particular, the E3N cohort is a well-educated population, with a low BMI, high levels of physical activity, and few current smokers. This means that the study may not be representative of the general population.

## Conclusion

Non-O blood groups were associated with an increased risk of dyslipidaemia. In the whole cohort, the association was specifically attributed to the A-group. No interaction was observed with smoking status. People of the A-blood group should be considered at high risk for dyslipidaemia, which could present as a group that may benefit from early intervention on cholesterol levels.

## Supplementary Information

Supplementary Information.

## Data Availability

The datasets used and/or analysed during the current study are available from the corresponding author on reasonable request.

## Abbreviations

BMI: Body mass index
MHT: Menopausal hormone therapy
