# Supplementary material for: Associations between smoking and blood-group, and the risk of dyslipidaemia amongst French women
Source: Sci Rep. 2021 Jul 21;11:14844. doi: 10.1038/s41598-021-94239-9 (PMC8295360; doi:10.1038/s41598-021-94239-9)

**Supplement**

Supplementary Table 1: Participant characteristics for those free of dyslipidaemia at the start of follow up depending on blood ABO group.

|  | O group (n = *20,260*) | Non-O group (n = *25,665*) | A-group (n = 19,899) | B-group (n = 4020) | AB-group (n = 1746) |
| --- | --- | --- | --- | --- | --- |
| Cholesterol | 5.4 (0.8) | 5.4 (0.8) | 5.4 (0.8) | 5.4 (0.8) | 5.5 (0.8) |
| Missing cholesterol data | 47.9 | 48.7 | 48.8 | 48.4 | 49.3 |
| Age at baseline (years), mean (SD) | 60.7 (6.3) | 60.7 (6.3) | 60.7 (6.3) | 60.8 (6.4) | 60.7 (6.2) |
| BMI (kg / m^2^), mean (SD) | 23.7 (3.7) | 23.7 (3.8) | 23.7 (3.8) | 23.7 (3.9) | 23.0 (3.8) |
| Total physical activity (Mets-h / week), mean (SD) | 61.9 (38.0) | 61.6 (37.6) | 61.6 (37.3) | 61.5 (37.0) | 61.8 (38.4) |
| Family history of cardiovascular disease (%) | 32.3 | 32.9 | 32.4 | 32.7 | 33.2 |
| Smoking (N / X / C) (%) | 52.5 / 37.1 / 10.4 | 52.6 / 37.1 / 10.3 | 52.7 / 37.0 / 10.3 | 52.8 / 36.7 / 10.4 | 50.7 / 39.3 / 10.0 |
| Prevalent hypertension (%) | 41.6 | 40.8 | 40.7 | 41.7 | 39.7 |
| Prevalent diabetes (%) | 2.8 | 3.0 | 3.0 | 3.2 | 3.6 |
| Education (high-school or higher) | 89.3 | 89.7 | 88.2 | 88.7 | 88.1 |
| Menopausal (%) | 89.1 | 88.0 | 87.8 | 88.7 | 88.5 |
| Age at menopause, mean (SD) | 50.6 (3.8) | 50.6 (3.8) | 50.6 (3.8) | 50.5 (3.9) | 50.6 (4.0) |
| Ever use of MHT (%) | 60.2 | 59.9 | 59.7 | 60.5 | 60.1 |

Abbreviations: BMI = body mass index, METs-h = Metabolic equivalent task-hours, N = never smoker, X = ex-smoker, C = current smoker, MHT = menopausal hormone therapy

Supplementary figure 1: Flowchart of inclusion/exclusion of the study participants, and analysis used for prevalent/incident cases.


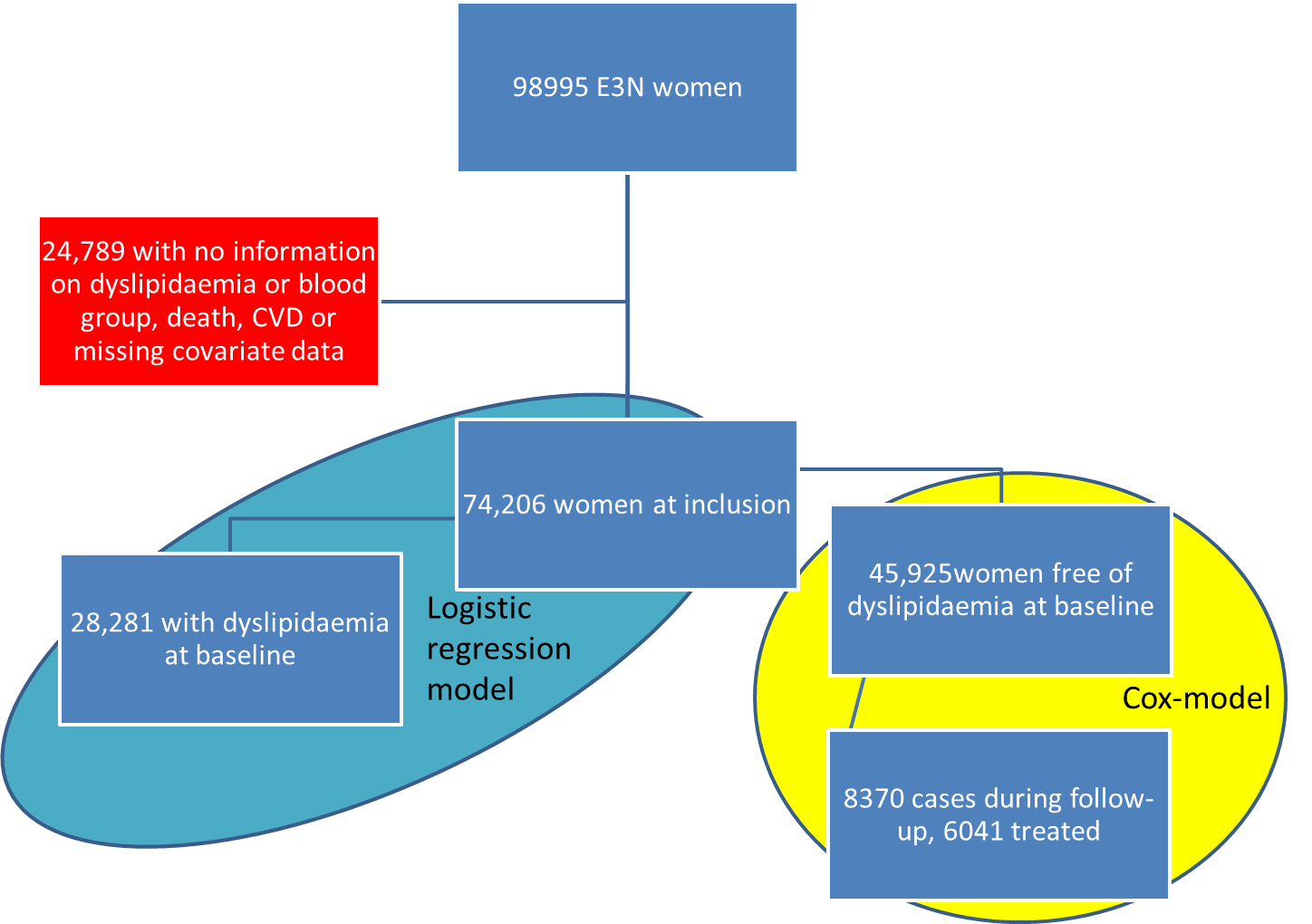

Supplement: Supplementary file 1 — Supplementary Information. [file 41598_2021_94239_MOESM1_ESM.docx]
